# Molecular mechanism analysis of m6A modification-related lncRNA-miRNA-mRNA network in regulating autophagy in acute pancreatitis

**DOI:** 10.1080/19382014.2022.2132099

**Published:** 2022-10-11

**Authors:** Xiang Li, Hong Qin, Ali Anwar, Xingwen Zhang, Fang Yu, Zheng Tan, Zhanhong Tang

**Affiliations:** a Critical Care Unit, the First Affiliated Hospital of Guangxi Medical University, Nanning, P.R. China; b Emergency Department (one), Hunan Provincial People’s Hospital, Changsha, Hunan, P.R. China; c Xiangya School of Public Health, Central South University, Changsha, P.R. China; d Food and Nutrition Society Gilgit Baltistan, Pakistan; e Emergency Department (three), Hunan Provincial People’s Hospital, Changsha, Hunan, P.R. China

**Keywords:** AP, autophagy, m6A modification, lncRNA, ceRNA, bioinformatics analysis

## Abstract

This study aims to explore the molecular mechanism of N6-methyladenosine (m6A) modification-related long noncoding RNA (lncRNA)–microRNA (miRNA)–messenger RNA (mRNA) network in regulating autophagy and affecting the occurrence and development of acute pancreatitis (AP). RNA-seq datasets related to AP were obtained from Gene Expression Omnibus (GEO) database and merged after batch effect removal. lncRNAs significantly related to m6A in AP, namely candidate lncRNA, were screened by correlation analysis and differential expression analysis. In addition, candidate autophagy genes were screened through the multiple databases. Furthermore, the key pathways for autophagy to play a role in AP were determined by functional enrichment analysis. Finally, we predicted the miRNAs binding to genes and lncRNAs through TargetScan, miRDB and DIANA TOOLS databases and constructed two types of lncRNA-miRNA-mRNA regulatory networks mediated by upregulated and downregulated lncRNAs in AP. Nine lncRNAs related to m6A were differentially expressed in AP, and 21 candidate autophagy genes were obtained. Phosphoinositide 3-kinase (PI3K)-Akt signaling pathway and Forkhead box O (FoxO) signaling pathway might be the key pathways for autophagy to play a role in AP. Finally, we constructed a lncRNA-miRNA-mRNA regulatory network. An upregulated lncRNA competitively binds to 13 miRNAs to regulate 6 autophagy genes, and a lncRNA-miRNA-mRNA regulatory network in which 2 downregulated lncRNAs competitively bind to 7 miRNAs to regulate 2 autophagy genes. m6A modification-related lncRNA Pvt1, lncRNA Meg3 and lncRNA AW112010 may mediate the lncRNA-miRNA-mRNA network, thereby regulating autophagy to affect the development of AP.

## Graphical Abstract

**Figure:**
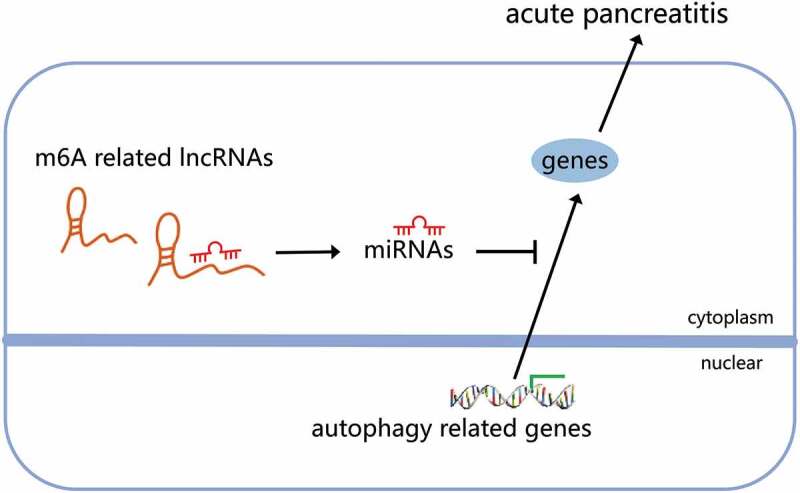

## Introduction

Acute pancreatitis (AP), an inflammatory disease of the pancreas with increasing global incidence, is the second-highest cause of total hospital stays, the most significant contributor to aggregate costs, and the fifth leading cause of in-hospital deaths.^1^ Although most patients have a mild course of AP and a mortality rate of less than 1%, 20% of AP will further develop into severe AP accompanied by systemic inflammatory response syndrome and the subsequent multiple organ dysfunction, including pancreatic necrosis, which caused approximately 10% of mortality.^2,3^ Furthermore, up to 18% of AP patients had a relapse, and about 8% of AP would further develop into chronic pancreatitis.^4^ Although great progress has been made in understanding AP, its pathogenesis has not been fully clarified.

The current paradigm is that pancreatitis initiates in injured acinar cells, the primary exocrine pancreas cell type.^5^ The data indicate that disordered acinar cell autophagy has been implicated in AP initiation.^6^ Autophagy is the major catabolic process by which cells eliminate damaged, defective, or unwanted cytoplasmic organelles, long-lived proteins, and lipids and recycle their constituents for energy and biogenesis needs.^7^ In particular, genetic ablation of the essential autophagy proteins in pancreatic epithelial cells caused spontaneous pancreatitis in mice.^8,9^ Collectively, maintenance of efficient autophagy in acinar cells plays an important protective role against the onset and progression of pancreatitis, but the specific mechanism is not very clear. Exploring the upstream regulation mechanism of autophagy-related proteins may be a meaningful direction.

Previous studies have shown that long noncoding RNA (lncRNA)–microRNA (miRNA)–messenger RNA (mRNA) network plays a role in the process of AP.^10,11^ The main way for lncRNA to play its role is to regulate the expression of autophagy genes through competing endogenous RNA (ceRNA) mechanisms.^12^ There is evidence that the expression level of lncRNAs is affected by N6-methyladenosine (m6A) modification, which plays important roles in physiological and pathological processes.^13–15^ As the new field of “RNA epigenetics” has been booming, m6A has been identified as a posttranscriptional regulatory mark in multiple RNA species, lncRNAs are included, and also, m6A is the most prevalent internal modification of RNA in eukaryotic cells.^16^ Cui et al. found that RNA m6A demethylase FTO-mediated epigenetic up-regulation of LINC00022 promotes tumorigenesis in esophageal squamous cell carcinoma.^17^ In addition, m6A is highly enriched on lncRNA THOR transcripts, the specific m6A readers YTHDF1 and YTHDF2 can read the m6A motifs and regulate the stability of the lncRNA THOR (stabilization and decay), and the internal m6A modification of the lncRNA THOR regulates the proliferation of cancer cells.^18^ Interestingly, m6A modification of circRNAs may participate in severe AP progression by regulating circRNA-miRNA networks.^19^ However, the lncRNA-mediated ceRNA regulatory network in AP is rarely studied. Therefore, exploring the lncRNA-miRNA-mRNA network mediated by m6A modification in AP may be an innovative direction.

So, based on the transcriptome RNA sequencing data set in Gene Expression Omnibus (GEO) database, we explored the molecular mechanism of m6A modification mediated lncRNA-miRNA-mRNA network regulating autophagy and affecting AP progression through bioinformatics analysis. This project aims to further explore the molecular mechanism of m6A modification and ceRNA network in regulating autophagy and provide new targets for diagnosing and treating AP.

## Materials and methods

### Data sources and downloads

From the GEO database (https://www.ncbi.nlm.nih.gov/gds), three RNA-seq datasets of pancreatic tissue from mice with AP were downloaded. GSE3644 contained three pancreatic tissue samples from control mice and three pancreatic tissue samples from mice with AP, GSE109227 contained four pancreatic tissue samples from control mice and four pancreatic tissue samples from mice with AP, and GSE121038 contained five pancreatic tissue samples from control mice and six pancreatic tissue samples from mice with AP. Due to the availability of public data in the GEO database, this study does not require ethical approval or informed consent.

### Multi-dataset merging and batch effect removal

Heterogeneity and latent variables are now widely recognized as major sources of bias and variability in high-throughput experiments. The most well-known source of latent variation in genomic experiments is batch effects - when samples are processed on different days, in different groups or by different people. The “sva” package supports surrogate variable estimation with the sva function, direct adjustment for known batch effects with the ComBat function.

In this study, three RNA-seq datasets GSE3644, GSE109227 and GSE121038 of pancreatic tissues of mice with AP were merged based on the common genes. Then, we used “sva” package to identify, estimate and remove unwanted sources of variation in the three datasets.

### Gene differential expression analysis and heatmap drawing

Limma is an R/Bioconductor software package that provides an integrated solution for analyzing data from gene expression experiments. Differentially expressed genes in the merged dataset were identified using the “limma” package in R software, with *p* < .05 as the threshold. Then, the heatmap was drawn by the “pheatmap” package in R software.

### Correlation analysis

Correlations between gene expression levels may indicate regulatory relationships. In order to clarify the association between m6A modification and lncRNA in AP, the correlation between m6A genes and lncRNAs expression was further analyzed based on the merged dataset. Pearson method was used to analyze the correlation between the expression of 15 m6A genes and 32 lncRNAs, with *p* < .05 set as the threshold. All analyses were performed by the “corrplot” package in R software.

### AP and autophagy-related gene retrieval

The search was performed by entering the search word “acute pancreatitis” in GeneCards database (https://www.genecards.org/) and CTD database (http://ctdbase.org/), take relevance score ≥10 and inference score ≥10 as the thresholds, respectively, and select the genes that meet the conditions. Enter the search word “autophagy” into the HADb Database (http://www.autophagy.lu/index.html) and download all Autophagy genes.

### Prediction of lncRNA-miRNA-mRNA binding relationship

The upstream miRNAs of the 21 candidate autophagy genes were predicted through TargetScan (http://www.TargetScan.org/vert_71/) and miRDB (http://www.miRDB.org/) databases. The screening standard of the TargetScan database was context ++ score ≤−0.20, and that of miRDB database was target score ≥80. The miRNAs binding to the nine candidate lncRNAs were predicted via DIANA TOOLS (http://diana.imis.athena-innovation.gr/DianaTools/index.php). The species selected in the above databases were limited to “mice.”

### Acquisition of intersected lncRNA/miRNA/gene

In this part, 28 lncRNAs were intersected with differentially expressed lncRNAs in AP to obtain candidate lncRNAs using jvenn tools (http://www.bioinformatics.com.cn/static/others/jvenn/example.html). The differentially expressed genes in AP were intersected with the screening results of GeneCards and CTD databases to obtain disease genes. The candidate autophagy genes were obtained by the intersection of disease genes and autophagy genes. In addition, the screening results of the TargetScan and miRDB database were intersected to obtain the targeted binding miRNAs of each candidate autophagy gene. Key miRNAs were obtained by the intersection of targeted binding miRNAs of candidate autophagy genes and miRNAs bound by lncRNAs.

### Gene functional enrichment analysis

GO and KEGG enrichment analyses were performed on 21 autophagy genes using the “clusterProfiler” package in R software, with *p* < .05 set as the threshold. GO consists of three parts: biological process (BP), cellular component (CC) and molecular function (MF). The top 10 items of the three parts were selected respectively for drawing. KEGG enrichment is displayed with *p* < .05 as significant enrichment, and the top 30 were selected for drawing.

### Network visualization

Based on the correlation between lncRNA and m6A gene expression, m6A-lncRNA networks of nine candidate lncRNAs and related m6A genes were constructed using Cytoscape software (v3.8.0) and displayed in circle graphs according to the degree value (the number of connections between each node and other nodes). The regulatory networks of upregulated or downregulated candidate autophagy genes and their targeted binding miRNAs were constructed, respectively, and lncRNA-miRNA-mRNA regulatory networks mediated by upregulated or downregulated lncRNAs were constructed, respectively.

## Results

### Bioinformatics technology screening process

**Figure 1.**
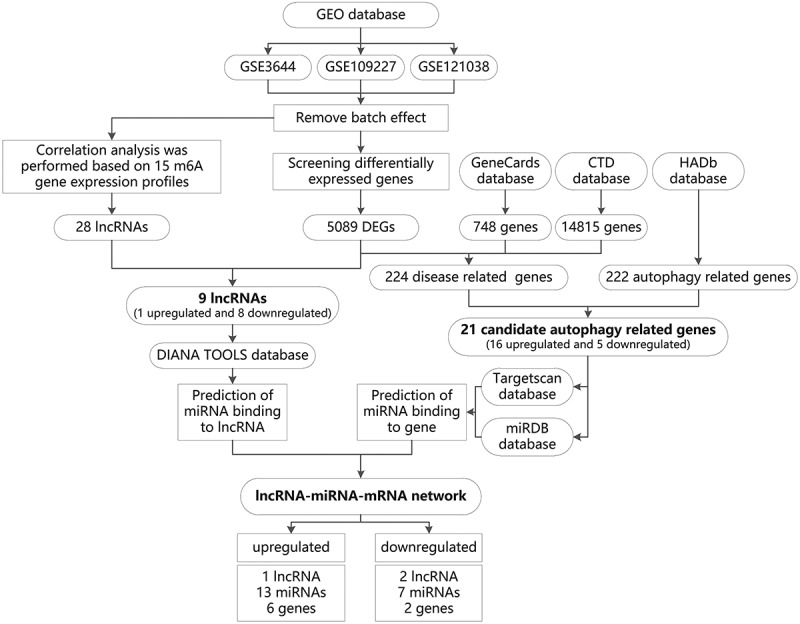
The flow chart of m6A modification-related lncRNA-miRNA-mRNA network in regulating autophagy and affecting the development of AP screened through bioinformatics technology.

Based on bioinformatics methods, this project screened the molecular mechanism of m6A modification-related lncRNA-miRNA-mRNA network in regulating autophagy and affecting the development of AP. The screening process is as follows: multiple datasets related to AP were retrieved in the GEO database and merged after batch effect removal. The expression profile of m6A was derived from the merged dataset, and the lncRNAs significantly related to m6A were screened through correlation analysis. The m6A-related lncRNAs differentially expressed in AP were further screened as candidate lncRNA. Furthermore, the differentially expressed genes in the merged dataset were intersected with the AP-related genes screened from GeneCards and CTD databases to obtain disease genes. The above disease genes were intersected with the autophagy genes obtained from autophagy-related databases to obtain candidate autophagy genes. GO and KEGG enrichment analyses were performed on candidate autophagy genes to determine key pathways for autophagy to play a role in AP. Finally, we categorized the candidate autophagy genes and candidate lncRNAs into two types: upregulation and downregulation. We predicted the common miRNAs through TargetScan, miRDB and DIANA TOOLS databases, and constructed an upregulated and a downregulated lncRNA-miRNA-mRNA regulatory network, respectively (Figure 1).

### Batch effect removal and data merging

**Figure 2.**
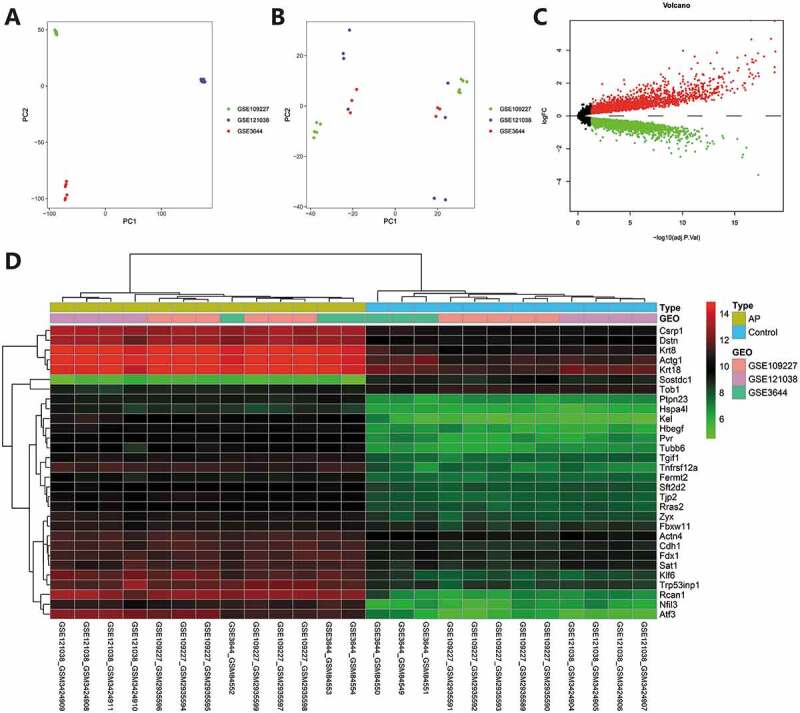
Multi-dataset merging and batch effect removal. (A) Sample distribution of three RNA-seq datasets before batch effect removal. A, sample distribution of three RNA-seq datasets before batch effect removal. (B) Sample distribution of three RNA-seq datasets after batch effect removal. (C) The volcano map of differential analysis of the merged dataset, red represents upregulation, green represents downregulation, black represents insignificant difference. (D) The heatmap of differential analysis of the merged datasets GSE3644 (control = 3, AP = 3), GSE109227 (control = 4, AP = 4), GSE121038 (control = 5, AP = 6).

To increase the sample data related to AP as much as possible and improve the accuracy of analysis results, we screened three RNA-seq datasets of pancreatic tissue of mice with AP through the GEO database, namely GSE3644, GSE109227 and GSE121038. The above three datasets were all AP models constructed through caerulein injection into C57BL6 mice, and the pancreatic tissue was used for transcriptome RNA-seq after 3–9 h. We used the combat function of “sva” package in R software to remove the batch effect. Before batch effect removal, the distribution of each sample data did not intersect (Figure 2A). After batch effect removal, each sample data was evenly cross-distributed, indicating a good batch effect removal (Figure 2B). After the three datasets were merged and the batch effect was removed, common genes were obtained and differential analysis was carried out with *p* < .05 as the threshold. As a result, 5089 differentially expressed genes were screened (Figure 2C), and the heatmap was drawn (Figure 2D). Subsequent screening of lncRNA and autophagy genes would be performed based on this merged dataset.

### Screening of m6A-related lncRNAs

**Figure 3.**
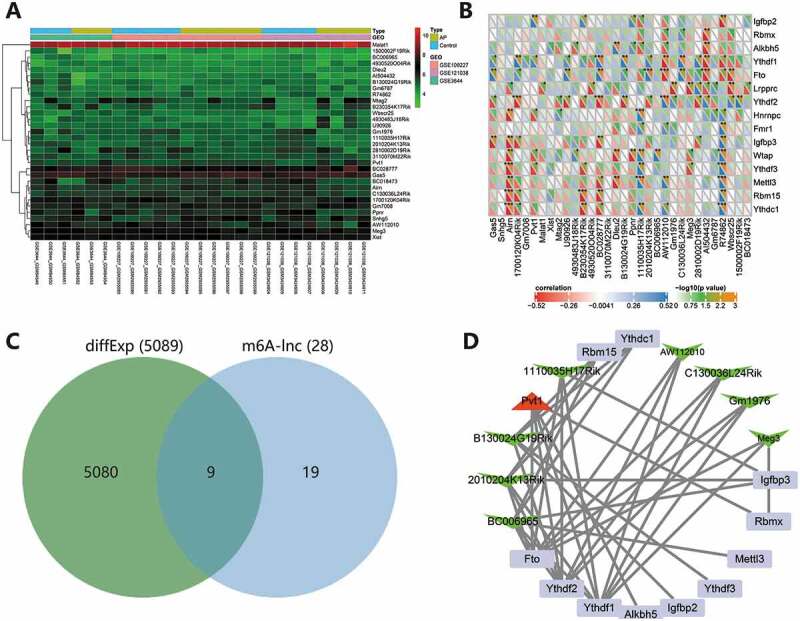
m6A-lncRNA network construction. (A) The expression heatmap of 32 lncRNAs in the merged dataset. (B) Correlation analysis of the expression of 15 m6A genes and 32 lncRNAs. *p < .05. **p < .01. ***p < .001. The X-axis indicates lncRNAs, and the *Y*-axis indicates m6A genes. (C) The Venn diagram of intersection of lncRNAs significantly related to m6A and differentially expressed genes in AP. (D) The m6A-lncRNA network of 9 candidate lncRNAs and 11 related m6A genes was constructed. The degree value gradually decreased clockwise from Ythdf1. Red represents upregulation, green represents downregulation, the square represents m6A genes, the triangle represents upregulated lncRNAs, and V-shape represents downregulated lncRNAs.

We screened the expression profiles of 15 known m6A genes from the merged dataset, namely *Mettl3*, *Wtap*, *Rbm15*, *Ythdc1*, *Ythdf1*, *Ythdf2*, *Ythdf3*, *Hnrnpc*, *Fmr1*, *Lrpprc*, *Igfbp2*, *Igfbp3*, *Rbmx*, *Fto* and *Alkbh5*. In addition, we screened the expression profiles of 32 lncRNAs from the merged dataset (Figure 3A). Pearson method was used to analyze the correlation between the expression of 15 m6A genes and 32 lncRNAs, with *p* < .05 set as the threshold. We screened 28 lncRNAs that were significantly correlated with m6A genes, and four lncRNAs with no significant correlation were deleted (Figure 3B). Further, the 28 lncRNAs were intersected with differentially expressed genes in AP to obtain 9 candidate lncRNAs (Figure 3C). Finally, we constructed a m6A-lncRNA network of the nine candidate lncRNAs and related m6A genes through Cytoscape software. The correlation analysis results are detailed in Supplementary Table 1. According to the arrangement of degree value (the number of connections between each node and other nodes), the lncRNAs related to m6A modification were BC006965, 2010204K13Rik, B130024G19Rik, Pvt1, 1110035H17Rik, AW112010, C130036L24Rik, Gm1976 and Meg3, successively. In addition, *Ythdf1*, *Ythdf2* and *Fto* may be the main genes affecting m6A modification of lncRNAs in AP (Figure 3D).

### Screening and functional enrichment analysis of autophagy genes

**Figure 4.**
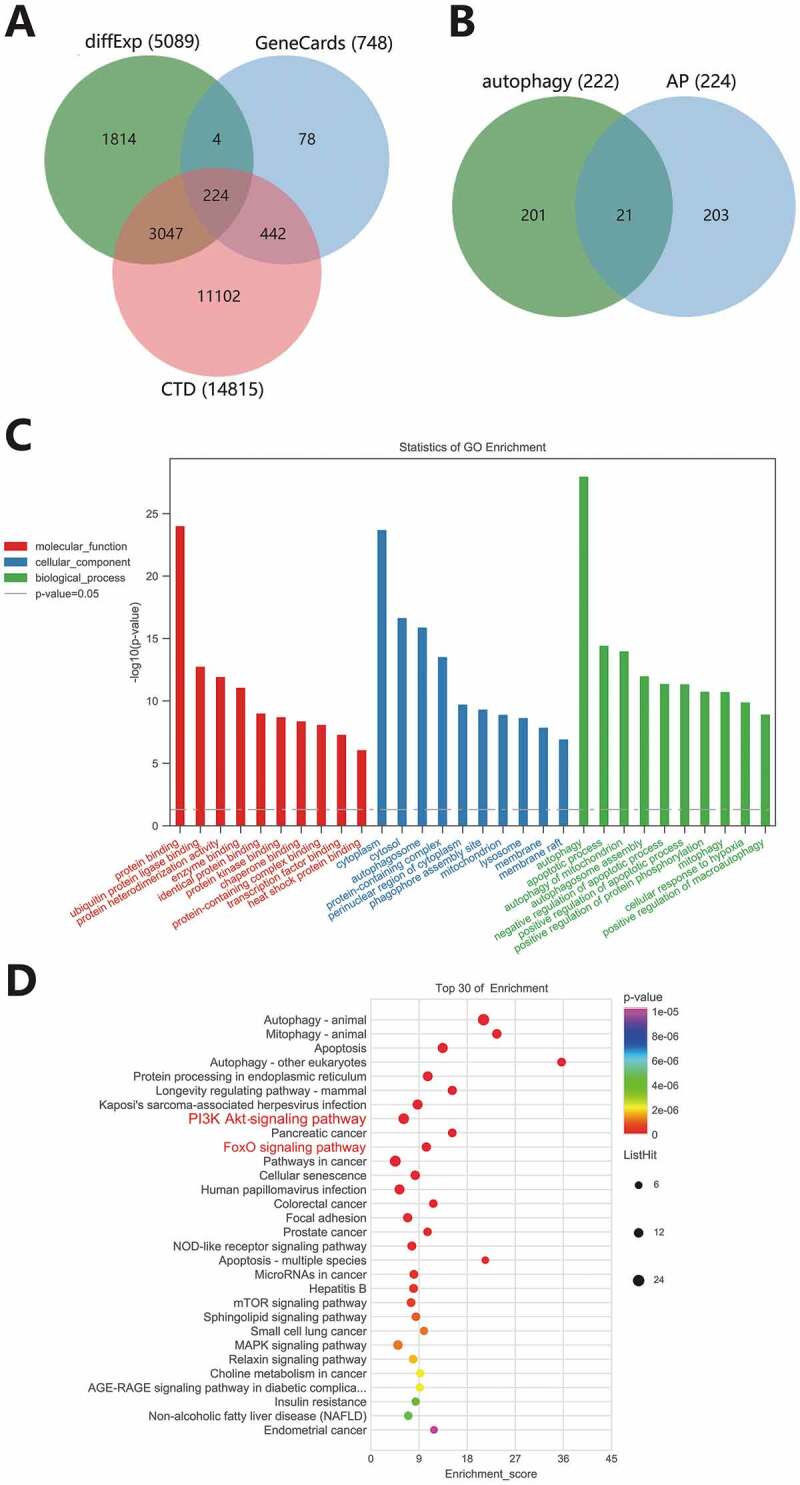
Functional enrichment analysis of autophagy genes. (A) The Venn diagram of intersection between differentially expressed genes in AP and screening results from GeneCards and CTD database. (B) The Venn diagram of intersection of disease genes and autophagy genes. C, GO-MF (red), GO-CC (blue) and GO-BP (green) analysis results of 21 autophagy genes. Column height indicates enrichment significance. (D) The KEGG analysis results of 21 autophagy genes. The color of the dots represents the *p*-value, and the dot size represents the number of enriched targets. The *X*-axis represents the number of enriched targets, and the *Y*-axis represents the pathway name.

Previously, we have screened 5089 differentially expressed genes in the merged dataset. We further searched for AP-related genes in combination with online databases. A total of 748 genes were screened in the GeneCards database with a relevance score ≥10 as the threshold, and 14815 genes were screened in the CTD database with an inference score ≥10 as the threshold. After the intersection of the above three, 224 disease genes were obtained (Figure 4A). A total of 222 autophagy genes were obtained from the HADb database, and 21 candidate autophagy genes were obtained via the intersection of 224 disease genes and 222 autophagy genes (Figure 4B). The association between the 21 candidate autophagy genes and the disease is shown in Supplementary Table 2.

We further analyzed the functional enrichment of the 21 candidate autophagy genes to identify the main pathways for autophagy to play a role in AP. The results of GO analysis showed that they were mainly enriched in MF such as protein binding (GO: 0005515), ubiquitin-protein ligase binding (GO: 0031625), protein heterodimerization activity (GO: 0046982), in CC such as cytoplast (GO: 0005737), cytosol (GO: 0005829) and autophagosome (GO: 0005776) and in BP including autophagy (GO: 0006914), apoptotic process (GO: 0006915) and autophagy of mitochondrion (GO: 0000422) (Figure 4C). KEGG analysis revealed that in addition to autophagy or apoptosis-related pathways (Figure 4D). These results further verified the representativeness of these 21 genes in autophagy.

In addition, the results of KEGG analysis also showed the top signaling pathways, with PI3K-Akt signaling pathway (path: mmu04151) and FoxO signaling pathway (path: mmu04068) ranking the top (Figure 4D). Combined with literature evidence, PI3K-Akt-mTOR pathway may be involved in AP progression by inhibiting autophagy.^20,21^ Nuclear FoxOs transactivate genes that control the formation of autophagosomes and their fusion with lysosomes. Independently of transactivation, cytosolic FoxO proteins induce autophagy by directly interacting with autophagy proteins.^22^ In general, we believe that the PI3K-Akt signaling pathway and FoxO signaling pathway may be the main pathways for autophagy genes to play a role in AP.

### Screening of upstream miRNAs of autophagy genes

**Figure 5.**
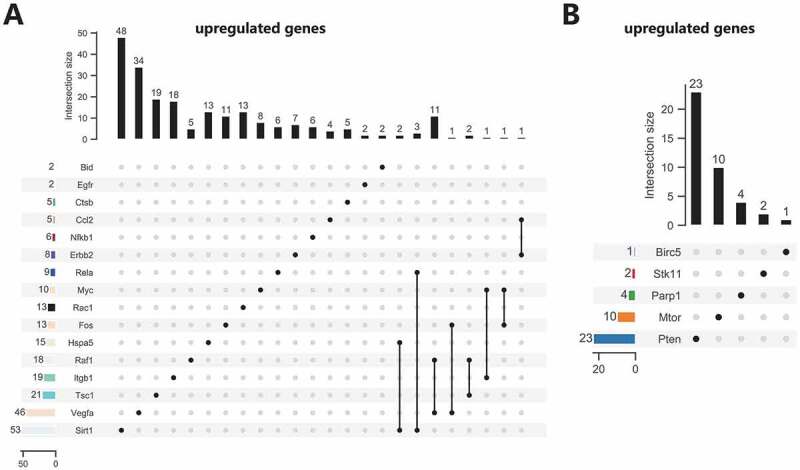
Screening of upstream miRNAs of the 21 candidate autophagy genes. (A) Upstream miRNA prediction of 16 upregulated candidate autophagy genes. (B) Upstream miRNA prediction of five downregulated candidate autophagy genes. The numerical value in the figure represents the predicted number of miRNAs, and the connecting line represents the jointly targeted miRNAs.

**Figure 6.**
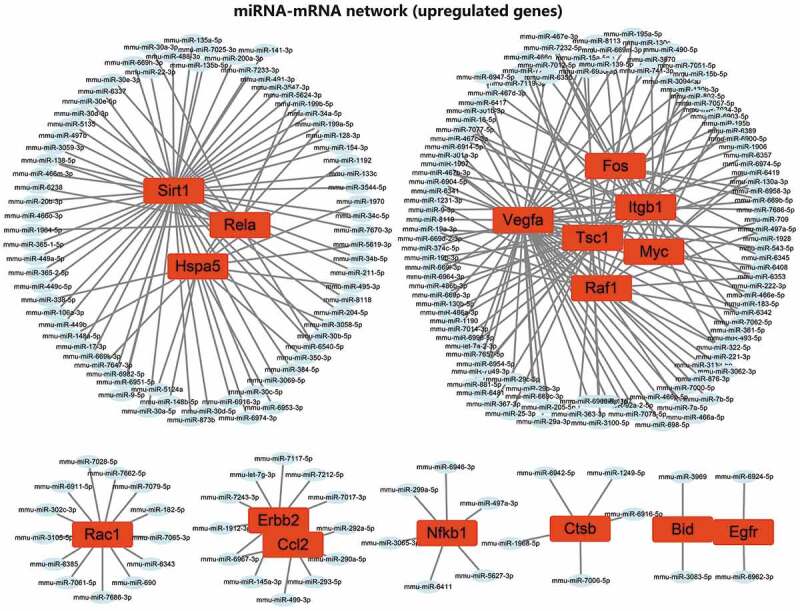
The regulatory network of 16 upregulated candidate autophagy genes and their targeted binding miRNAs. Red represents upregulation, the square represents genes, the ellipse represents miRNAs.

**Figure 7.**
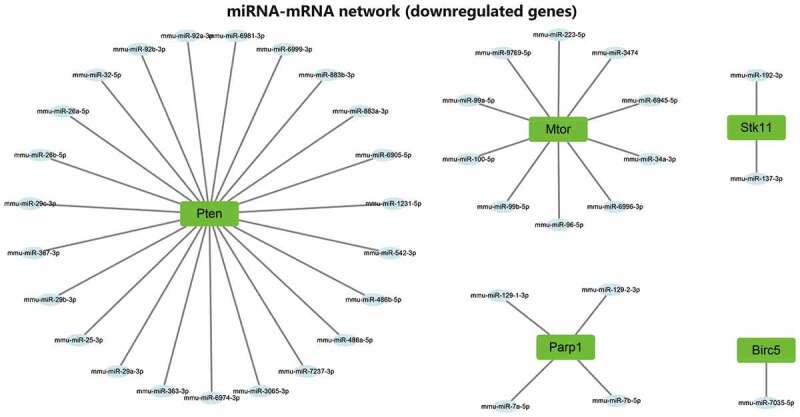
The regulatory network of 5 downregulated candidate autophagy genes and their targeted binding miRNAs. Green represents downregulation, the square represents genes, the ellipse represents miRNAs.

We then predicted miRNAs binding to 21 candidate autophagy genes through online databases. The screening standard of the TargetScan database was context ++ score ≤−0.20, and that of miRDB database was target score ≥80. The prediction results of TargetScan and miRDB databases were intersected to obtain the miRNAs bound by each autophagy gene. The 21 candidate autophagy genes were grouped into 16 upregulated and 5 downregulated genes, shown separately in Figure 5. Furthermore, the miRNA-mRNA networks were constructed for upregulated and downregulated genes and their targeted binding miRNAs through Cytoscape software. The results showed that Sirt1, Hspa5 and Rela had commonly targeted binding miRNAs in the miRNA-mRNA network constructed using upregulated autophagy genes. Vegfa, Tsc1, Itgb1, Fos, Myc and Raf1 shared a regulatory network in the same regulatory network, and ErbB2 and Ccl2 shared a regulatory network (Figure 6). There was no common targeted binding miRNA in the miRNA-mRNA network constructed by downregulated autophagy genes, and PTEN could target bind to the largest number of miRNAs (Figure 7).

### Construction of m6A related lncRNA-mediated lncRNA-miRNA-mRNA network regulating autophagy in AP

**Figure 8.**
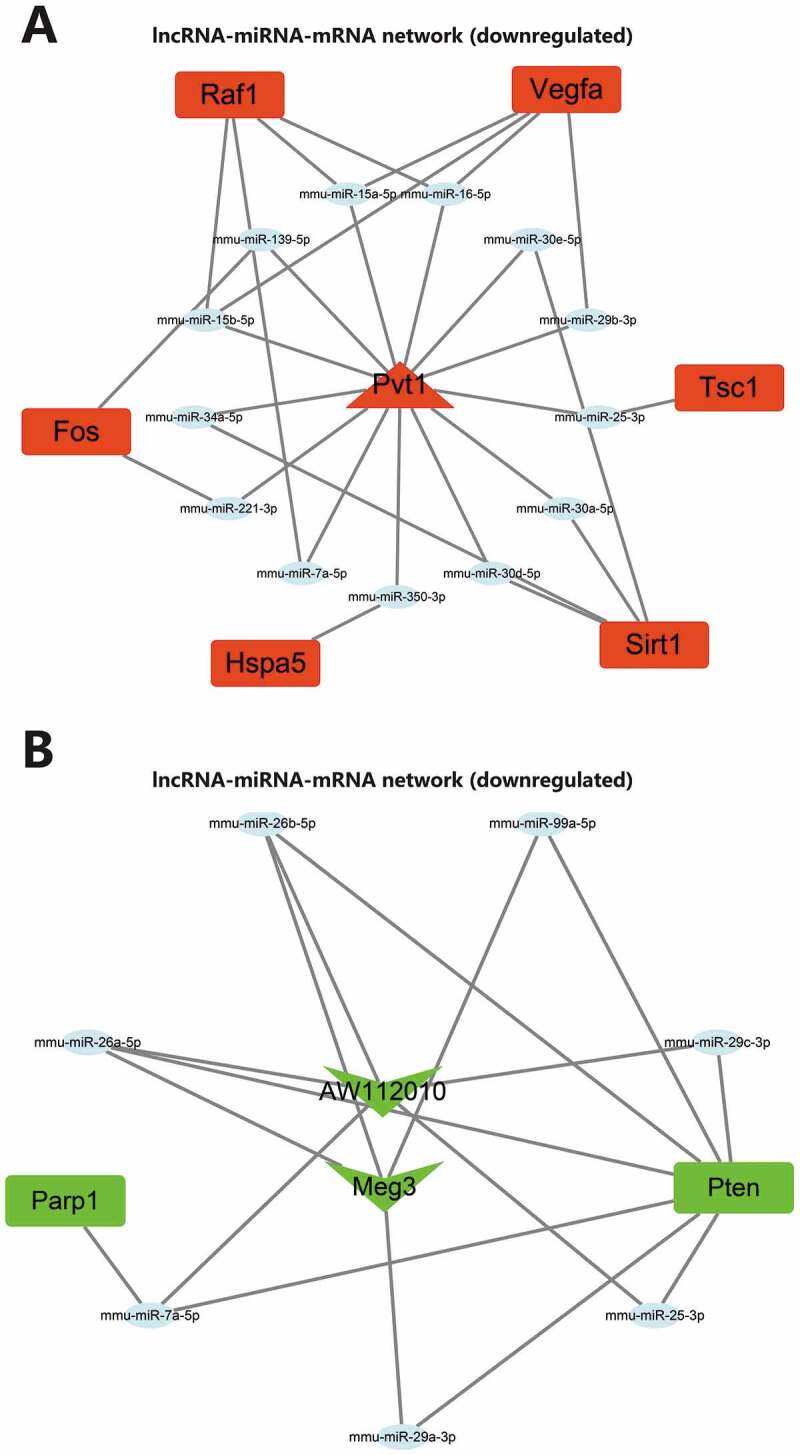
lncRNA-miRNA-mRNA network construction. (A) Upregulated lncRNA-mediated lncRNA-miRNA-mRNA regulatory network. (B) Downregulated lncRNA-mediated lncRNA-miRNA-mRNA regulatory network. In the figure, red represents upregulation, green represents downregulation, the square represents genes, the ellipse represents miRNAs, the triangle represents upregulated lncRNAs, and V-shape represents downregulated lncRNAs.

Similar to candidate autophagy genes, nine candidate lncRNAs were also categorized into one upregulated lncRNA and eight downregulated lncRNAs. miRNAs binding to the lncRNAs were predicted through the DIANA TOOLS database. The upregulated miRNAs and miRNAs predicted by upregulated autophagy genes were intersected, and the downregulated miRNAs and miRNAs predicted by downregulated autophagy genes were intersected. The lncRNA-miRNA-mRNA regulatory networks mediated by the upregulated and downregulated lncRNAs were constructed, respectively (Figure 8). So far, we believe that m6A modification may mediate the upregulation of lncRNA Pvt1 expression in AP. Through competitive binding with mmu-miR-139-5p, mmu-miR-15a-5p, mmu-miR-15b-5p, mmu-miR-16-5p, mmu-miR-221-3p, mmu-miR-25-3p, mmu-miR-29b-3p, mmu-miR-30a-5p, mmu-miR-30d-5p, mmu-miR-30e-5p, mmu-miR-34a-5p, mmu-miR-350-3p and mmu-miR-7a-5p, lncRNA Pvt1 could remove the targeted inhibitory effects of the miRNAs on Tsc1, Raf1, Sirt1, Hspa5, Vegfa and Fos. m6A modification in AP may mediate the downregulation of lncRNA Meg3 and lncRNA AW112010 expression. Through competitive binding to mmu-miR-25-3p, mmu-miR-26a-5p, mmu-miR-26b-5p, mmu-miR-29c-3p, mmu-miR-7a-5p, mmu-miR-29a-3p and mmu-miR-99a-5p, lncRNA Meg3 and lncRNA AW112010 could remove the targeted inhibitory effects of the miRNAs on Parp1 and Pten. The above two ceRNA regulatory networks may further regulate autophagy, affecting the disease process.

## Discussion

AP is one of the first pathological processes where autophagy has been described in human tissue. Here, we found that the PI3K-Akt signaling pathway and FoxO signaling pathway may be the main pathways for autophagy genes to play a role in AP. And m6A modification-related lncRNA Pvt1, lncRNA Meg3 and lncRNA AW112010 may mediate the lncRNA-miRNA-mRNA network, thereby regulating autophagy to affect the development of AP.

Bioinformatics is a new subject of genetic data collection, analysis and dissemination to the research community. Through the comprehensive utilization of biology, computer science, and information technology, bioinformatics reveals the biological mysteries endowed by many complex biological data, mainly focusing on genomics and proteomics. Bioinformatics analysis has been applied to the research of various diseases to reveal the possible molecular mechanism of the disease process and screen disease diagnosis, treatment and prognostic markers.^23–26^ For example, Gan et al. explored the clinical value and prospective pathway signaling of microRNA-375 in lung adenocarcinoma based on the cancer genome atlas (TCGA), GEO and bioinformatics analysis.^27^ More importantly, many studies have obtained key miRNAs, genes or pathways through bioinformatics analysis, verified in subsequent experiments.^28–30^ Such as, through Gene Set Enrichment Analysis (GSEA) and the co-expression network establishment, further quantitative reverse transcription-polymerase chain reaction (RT-PCR) analysis contributed to examining the expression levels of lncRNA TINCR, miR-107 and CD36, then the dual luciferase assay was used to validate the association between miR-107 and lncRNA TINCR or CD36. Finally, a regulatory function of the lncRNA TINCR/miR-107/CD36 axis in CRC was revealed.^31^ Therefore, this project explored the ceRNA mechanism upstream of autophagy-related genes through bioinformatics analysis methods and constructed lncRNA-miRNA-mRNA network, which provides a significant reference value for subsequent in vivo and in vitro experiments.

A total of 21 candidate autophagy genes were screened in this project. The gene function enrichment analysis results showed that the above candidate autophagy genes were mainly enriched in PI3K-Akt and FoxO signaling pathways. The study found that abdominal paracentesis drainage attenuates severe AP by enhancing cell apoptosis via the PI3K-Akt signaling pathway.^32^ Wortmannin, PI3K-Akt signaling pathway inhibitor, attenuates severe AP in rats.^33,34^ Besides, FoxO emerges as a key factor for maintaining a functional endocrine pancreas. The FoxO/Bcl6/cyclin D2 pathway regulates cell cycle control in pancreatic beta-cells.^35^ And a constitutively active form of FoxO3 also induced autophagy, suggesting FoxO3 as a downstream target of the PI3K pathway for autophagy inhibition.^36^ Based on the above evidence, we believe that PI3K-Akt and FoxO signaling pathways may be the main pathways of the autophagy gene in AP.

Recently, the new field of “RNA epigenetics” has been booming, and N6-methyladenosine (m6A) has been identified as a posttranscriptional regulatory mark in multiple RNA species.^37^ The evidence showed that abnormal m6A methylation plays a significant role in the process of numerous diseases,^38–40^ and m6A modification of circRNAs may be involved in the pathogenesis of severe AP.^19^ Therefore, we speculate that m6A modification of lncRNA may also play a role in the AP progression. This study proposed for the first time that m6A modification in AP may mediate the upregulation of lncRNA Pvt1 expression and the downregulation of lncRNA Meg3 and lncRNA AW112010 expression. Consistent with the above, other studies have also found that m6A modification of lncRNA Pvt1 governs epidermal stemness,^41^ and ALKBH5-mediated m6A modification of lncRNA Pvt1 plays an oncogenic role in osteosarcoma.^42^ In addition, m6A-induced lncRNA Meg3 suppresses hepatocellular carcinoma cell’s proliferation, migration, and invasion.^43^ However, no studies have shown that m6A modification of lncRNA AW112010 plays a role in the pathological process, which is the direction we can continue to explore.

ceRNA are transcripts that can regulate each other at the post-transcription level by competing for shared miRNAs. ceRNA networks link the function of protein-coding mRNAs with non-coding RNAs such as microRNA, long non-coding RNA, pseudogenic RNA and circular RNA.^44^ Some progress has been made in the research of ceRNA in AP.^10,11^ In this study, ceRNA regulatory networks of three lncRNAs have been constructed to clarify the downstream key genes, which also connect the function of lncRNA and autophagy. We found that through ceRNA regulatory networks, lncRNA Pvt1 may regulate the expression of Tsc1, Raf1, Sirt1, Hspa5, Vegfa and Fos, lncRNA Meg3 and lncRNA AW112010 may regulate the expression of Parp1 and Pten. These key genes may be important targets to intervene in autophagy in AP.

Tsc1 and other genes are closely related to autophagy and play a certain role in AP. Mechanistically, Tsc1 deficiency led to autophagy suppression^45^ and a direct connection between Raf-1 activation and cellular autophagy.^46^ Rong Y et al. found that resveratrol can protect rats against SAP by activating the Sirt1-FoxO1 axis,^47^ consistent with the key pathways found in this project. The activation of the Sirt1-autophagy signaling pathway alleviates AP.^48^ Research shows that more severe chronic pancreatitis is associated with significantly increased Hspa5 mRNA levels.^49^ Crosstalk between Hspa5 arginylation and sequential ubiquitination leads to AKT degradation through autophagy flux.^50^ In addition, the increased immunohistochemical expression of Vegfa can play an important role in tracking the evolution and pathology of AP.^51^ Several studies have shown that Vegfa is closely related to autophagy.^52–54^ Overexpression of c-Fos increased the Beclin1-induced autophagy.^55^ Ultimately, Parp1 is a key regulator of cell death. Its inhibition prevented streptozotocin-induced diabetes and attenuated caerulein-induced AP.^56^ The targeted inhibition of Pten expression in AP can induce acinar cell apoptosis and inhibit inflammatory response.^57^ Pten phosphorylation promotes its nuclear translocation and autophagy, but the role of Pten in acinar cell autophagy is not clear.^58^ In general, some studies have shown the potential function of the above key genes in AP.

In addition to the above eight genes (*Tsc1*, *Raf1*, *Sirt1*, *Hspa5*, *Vegfa*, *Fos*, *Parp1* and *Pten*) in the ceRNA regulatory network, other autophagy-related genes also have certain functions in AP, but may not be affected by m6A modification, nor through the regulation of ceRNA. For example, inhibition of Rac1 decreases the severity of pancreatitis and pancreatitis-associated lung injury in mice.^59^ Cathepsin B (Ctsb) aggravates AP by activating the NLRP3 inflammasome and promoting the caspase-1-induced pyroptosis.^60^ mTOR-Myc axis drives acinar-to-dendritic cell transition and the CD4+ T cell immune response in AP.^61^ More importantly, the three key lncRNAs may also combine with other miRNAs to regulate the expression of autophagy genes and participate in the occurrence and development of AP. Hu et al. Found lncRNA Pvt1 aggravates severe AP by promoting autophagy via the miR-30a-5p/Beclin-1 axis.^62^ lncRNA Meg3 participates in caerulein-induced inflammatory injury in human pancreatic cells via regulating miR-195-5p/FGFR2 axis and inactivating NF-kappaB pathway.^63^ The above evidence shows that although the conclusion of this study lacks in vitro and in vivo experimental verification which is a certain limitation, it is obvious that the ceRNA regulatory network constructed in this project also references future mechanism research and brings good theoretical guidance value.

Based on the above evidence, we conclude that m6A modification-related lncRNA Pvt1, lncRNA Meg3 and lncRNA AW112010 may mediate the lncRNA-miRNA-mRNA network to regulate autophagy and further affect the development of AP. This provides new clues for further exploring the specific mechanism of the occurrence and development of AP and provides a new theoretical basis for screening markers for the diagnosis and treatment of AP. The following work will further carry out experiments in vivo and in vitro to verify the above molecular mechanisms.
